# The influence of body mass index on the tolerability and effectiveness of full-weight-based paclitaxel chemotherapy in women with early-stage breast cancer

**DOI:** 10.1007/s10549-022-06681-6

**Published:** 2022-08-16

**Authors:** Lishi Lin, Marcel Soesan, Dorieke E. M. van Balen, Jos H. Beijnen, Alwin D. R. Huitema

**Affiliations:** 1 Department of Pharmacy and Pharmacology, The Netherlands Cancer Institute-Antoni Van Leeuwenhoek Hospital, Amsterdam, The Netherlands; 2 Department of Internal Medicine, The Netherlands Cancer Institute-Antoni Van Leeuwenhoek Hospital, Plesmanlaan 121, 1066 CX Amsterdam, The Netherlands; 3 Department of Pharmaceutical Sciences, Utrecht University, Utrecht, The Netherlands; 4 Department of Pharmacology, Princess Máxima Center for Pediatric Oncology, Utrecht, The Netherlands; 5 Department of Clinical Pharmacy, University Medical Center Utrecht, Utrecht University, Utrecht, The Netherlands

**Keywords:** Obesity, Body mass index, Paclitaxel, Chemotherapy, Relative dose intensity, Breast cancer

## Abstract

**Purpose:**

To investigate the influence of body mass index (BMI) on the tolerability and effectiveness of full-weight-based paclitaxel chemotherapy in early breast cancer patients.

**Methods:**

Early-stage breast cancer patients who received (neo)adjuvant weekly paclitaxel 80 mg/m^2^ chemotherapy were included in this retrospective study. Patients were divided into three groups based on their BMI: lean, overweight, and obese. Logistic regression was used to assess for association between BMI with administered relative dose intensity (RDI) < 85%. The occurrence of treatment modifications and the pathological response on neoadjuvant chemotherapy were compared between BMI categories.

**Results:**

Four hundred (400) patients were included in this study; 200 (50%) lean, 125 (31%) overweight, and 75 (19%) obese patients. The adjusted odds ratio to receive RDI < 85% for BMI was 1.02 (p value, .263). Treatment modifications occurred in 115 (58%), 82 (66%), and 52 (69%) patients in the respective BMI categories (p value = .132). Peripheral neuropathy was observed in 79 (40%), 58 (46%), and 41 (55%) patients in the lean, overweight, and obese group (p value = .069), whereas hematologic toxicity was observed in 31 (16%), 10 (8%), and 4 (5%) patients (p value = .025). Pathological complete response was observed in 22 (17%), 11 (14%), and 6 (13%) patients in the respective BMI categories (p value = .799).

**Conclusion:**

BMI did not significantly influence the tolerability and effectiveness of full-weight-based paclitaxel chemotherapy. Therefore, the results of this study align with current guideline recommendations of using full-weight-based paclitaxel chemotherapy in obese patients.

## Introduction

In recent decades there is an immense rise in prevalence of overweight and obesity. According to the World Health Organization more than 650 million adults are obese, which is 13% of the world population [1]. Obesity is a strong predictor of worse health outcomes in different diseases and can also complicate drug dosing [2, 3].

For chemotherapy, it was common use to empirically lower the full-weight-based dosage in obese patients, which is also known as dose capping [4]. One of the reasons to perform dose capping in obese patients is to prevent excessive toxicity [5]. However, there was no evidence that toxicity was increased among obese patients receiving full-weight-based chemotherapy doses, while some studies do suggest compromised survival outcomes due to dose capping [4, 6–11]. Therefore, a clinical practice guideline was released in 2012 by the American Society of Clinical Oncology (ASCO) recommending to use full-weight-based chemotherapy doses in obese patients [12]. As this guideline has been updated last year with additional recommendations for immunotherapy and targeted therapy dosing, the recommendations for chemotherapy dosing remained unchanged [13].

A recent study in early breast cancer patients showed that obese patients treated with adjuvant docetaxel-containing chemotherapy received a lower relative dose intensity (RDI) compared to lean patients, while no upfront dose capping was applied in obese patients due to excess weight. In addition, obese patients had a shorter disease-free survival and overall survival compared to lean patients, which was also the case when only patients with a RDI of 85% or higher were analyzed. Therefore, it appears that obese patients tolerate full-weight-based docetaxel chemotherapy less well compared to lean patients, which negatively influences survival outcomes. These trends were not observed in patients who received non-docetaxel chemotherapy, suggesting a differential response to docetaxel according to body mass index (BMI) [14].

As obesity has become increasingly common and adjuvant chemotherapy with taxanes in early breast cancer is a cornerstone of the treatment, it is important to study whether these findings for docetaxel are also observed for paclitaxel chemotherapy. Therefore, the aim of this study was to investigate the influence of BMI on the tolerability and effectiveness of full-weight-based paclitaxel chemotherapy in early breast cancer patients.

## Patients and methods

This retrospective observational cohort study was conducted at the Netherlands Cancer Institute-Antoni van Leeuwenhoek hospital (NKI-AvL), Amsterdam, the Netherlands. Patients diagnosed with early breast cancer between January 2016 and July 2020, who received paclitaxel 80 mg/m^2^ in a weekly regimen as (neo)adjuvant treatment were included in this study. Only patients with estrogen receptor-positive (ER+) and/or human epidermal growth receptor 2-positive (HER2+) breast cancer were included. Patients with triple-negative breast cancer were excluded as they receive a combination of paclitaxel and carboplatin at our institute. Patients with ER+/Her2− breast cancer received doxorubicin 60 mg/m^2^ with cyclophosphamide 600 mg/m^2^ every 2 or 3 weeks for four cycles followed by paclitaxel 80 mg/m^2^ weekly for 12 cycles. Patients with cT1N0 Her2+ breast cancer received paclitaxel 80 mg/m^2^ weekly for 12 cycles as chemotherapy, in combination with trastuzumab with a loading dose of 300 mg, followed by 150 mg every week during the paclitaxel chemotherapy. Trastuzumab treatment in these patients continued up to 1 year, in which 450 mg was administered every 3 weeks. Before August 2017, a loading dose of 4 mg/kg trastuzumab was used, whereas during following cycles a dose of 2 mg/kg was administered every week, followed by 6 mg/kg trastuzumab every other 3 weeks up to 1 year.

Data on patient characteristics, tumor characteristics, treatments, and toxicity were extracted from the electronic medical record (EMR) HiX (ChipSoft, Amsterdam, the Netherlands). The conduct of this observational study was approved by the Investigational Review Board of the NKI-AvL and the need for written informed consent was waived.

Patients were categorized into three groups based on their BMI according to the categories of the World Health Organization: underweight (< 18.5 kg/m^2^), lean (≥ 18.5 and < 25 kg/m^2^), overweight (≥ 25 and < 30 kg/m^2^), and obese (≥ 30 kg/m^2^). Underweight patients were excluded from this study due to a low frequency (n = 3). BMI for each patient was calculated as the median of all BMI measurements during the course of chemotherapy treatment. Chemotherapy doses for each patient could change during the course of chemotherapy treatment according to weight changes and were determined by calculating the body surface area (BSA) using the formula of DuBois [15].

Study outcomes were the occurrence of dose capping and the median-administered RDI. In addition, it was determined how often treatment modifications occurred and what toxicity caused these modifications. For patients who received neoadjuvant chemotherapy, the pathological response of the tumor to chemotherapy treatment was also assessed as a surrogate endpoint for effectiveness.

RDI is calculated using the following formula:

$${\text{RDI}}\, = \,\frac{{{\text{Delivered}}\,{\text{total}}\,{\text{dose}}\,({\text{mg}})}}{{{\text{Time}}\,{\text{to}}\,{\text{complete}}\,{\text{the}}\,{\text{chemotherapy}}\,{\text{with}}\,{\text{imputation}}\,{\text{for}}\,{\text{missed}}\,{\text{cycles}}\,({\text{days}})}} \times \frac{{{\text{Standard}}\,{\text{time}}\,{\text{to}}\,{\text{complete}}\,{\text{the}}\,{\text{chemotherapy}}\,({\text{days}})}}{{{\text{Standard}}\,{\text{total}}\,{\text{dose}}\,({\text{mg}})}} \times 100\% .$$

The standard total dose was the amount of drug patients would receive if all chemotherapy courses were administered according to the calculated BSA. Time to complete the chemotherapy was defined as the time from the first day of the first cycle until the last day of the last cycle. The standard time to complete the described paclitaxel chemotherapy regimen was 84 days. If less than 12 cycles were administered and the determined time to complete the chemotherapy was shorter than 84 days, this was increased to 84 days as imputation for missed cycles.

Descriptive statistics were reported using frequencies for categorical data and means for continuous data. Categorical variables between BMI categories were compared with Fisher’s exact test. Continuous variables between BMI categories were compared using the ANOVA test or the Kruskal–Wallis test, depending on the appropriateness of the tests. Multivariable logistic regression was performed to assess association between BMI and administered RDI < 85% while adjusting for confounders. Two-sided p values < .05 were considered significant. All statistical analyses were performed in R version 4.0.4 (R Foundation for Statistical Computing, Vienna, Austria).

## Results

### Characteristics of the study population

In total 400 women were included in this study. Patient characteristics are depicted in Table 1. Of the patient population, 200 (50%) patients were lean, 125 (31%) patients were overweight, and 75 (19%) patients were obese. Variation of body weight during chemotherapy treatment was limited. The relative standard deviation of BMI within patients varied from 0 to 9%. Only five of the 400 patients had a relative standard deviation above 5%. Patients who were overweight or obese were statistically significantly older and more frequently postmenopausal as compared to lean patients.

**Table 1:** Characteristics of patients divided by BMI category

	Lean n = 200 (%)	Overweight n = 125 (%)	Obese n = 75 (%)	p value
Age, years				
Mean (SD)	48.8 (11.8)	53.1 (9.9)	54.5 (10.5)	< .001
Tumor size, cm				
< 2	92 (46)	54 (43)	28 (37)	.428
≥ 2	108 (54)	71 (57)	47 (63)	
No. of positive nodes				
0	132 (66)	82 (66)	44 (59)	.677
1–3	60 (30)	39 (31)	26 (35)	
≥ 4	8 (4)	4 (3)	5 (6)	
ER status				
Negative	14 (7)	11 (9)	4 (5)	.666
Positive	186 (93)	114 (91)	71 (95)	
PgR status				
Negative	62 (31)	45 (36)	18 (24)	.218
Positive	138 (69)	80 (64)	57 (76)	
HER2 status				
Negative	159 (80)	95 (76)	66 (88)	.113
Positive	41 (20)	30 (24)	9 (12)	
Histologic grade				
1	15 (7)	7 (6)	4 (5)	.756
2	115 (58)	70 (56)	40 (54)	
3	65 (33)	46 (37)	31 (41)	
Unknown	5 (2)	2 (1)	–	
Ki-67				
< 15%	70 (35)	38 (30)	22 (29)	.835
≥ 15%	114 (57)	78 (63)	48 (64)	
Unknown	16 (8)	9 (7)	5 (7)	
Smoking status				
Never	110 (55)	79 (63)	42 (56)	.190
Ever	90 (45)	46 (37)	32 (43)	
Unknown	–	–	1 (1)	
Menopausal status				
Premenopausal	123 (62)	58 (47)	34 (45)	.004
Postmenopausal	77 (38)	65 (52)	39 (52)	
Unknown	–	2 (1)	2 (3)	

### Relative dose intensity

**Fig. 1:**
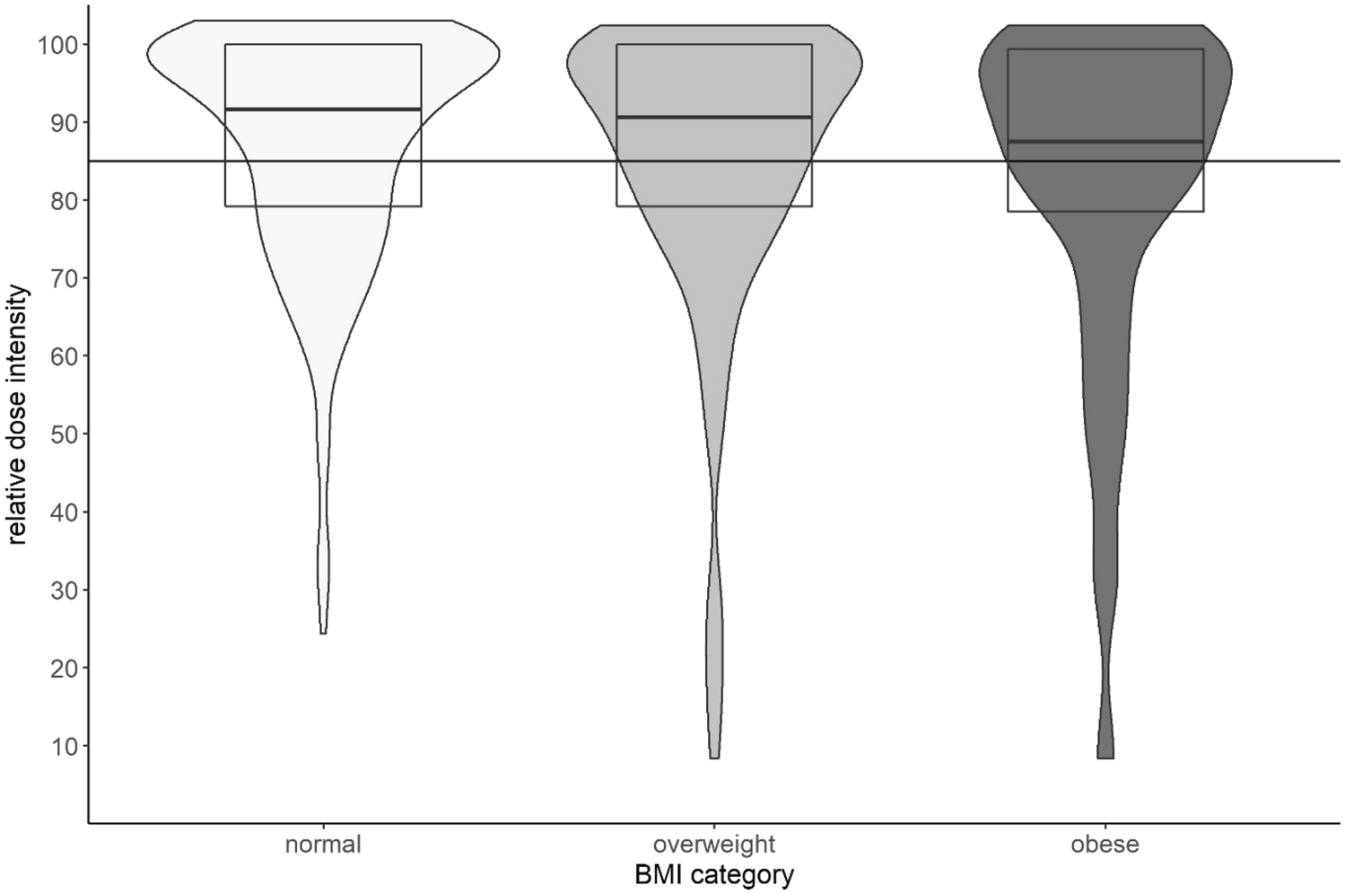
Administered relative dose intensity (RDI) for each body mass index (BMI) category visualized using a boxplot and a violin plot showing the distribution of RDI within each BMI category. The horizontal line shows a RDI of 85%

No patients had their chemotherapy dose capped due to excess weight. Patients in the lean, overweight, and obese group received a median RDI of 92%, 91%, and 88%, respectively. The distributions of administered RDI for the different BMI categories are shown in Fig. 1. The number of patients receiving a RDI < 85% were 68 (34%), 46 (37%), and 32 (43%) in the lean, overweight, and obese group, respectively. The univariable and multivariable logistic regression models assessing for association between BMI and administered RDI < 85% are shown in Table 2. The adjusted odds ratio (OR) for BMI was 1.02 (95% confidence interval (95% CI), 0.98–1.07) with a p value of .263. For a five-point difference for BMI, this translates to an adjusted OR of 1.13 (95% CI, 0.91–1.40) with a p value of .263.

**Table 2:** Univariable and multivariable logistic regression models examining association between body mass index and administered relative dose intensity < 85%

	Univariable			Multivariable		
	OR	95% CI	P value	OR	95% CI	P value
Body mass index	1.04	1.00–1.08	.051	1.02	0.98–1.07	.263
Age	1.04	1.02–1.06	< .001	1.04	1.02–1.06	< .001
Menopausal status, postmenopausal	2.00	1.32–3.03	.001			

*OR* odds ratio, *CI* confidence interval

### Toxicity

Treatment modifications occurred in 115 (58%), 82 (66%), and 52 (69%) patients in the lean, overweight, and obese group, respectively (p value = .132). Treatment modifications were divided into dose reductions and dose delays or cancellations. Dose reductions occurred in 87 (44%), 60 (48%), and 36 (48%) patients in the lean, overweight, and obese group, respectively (p value = .676). Dose delays or cancellations occurred in 82 (41%), 59 (47%), and 34 (45%) patients, in the respective BMI categories (p value = .528).

The most common types of toxicities leading to treatment modifications were peripheral neuropathy and hematologic toxicity. Peripheral neuropathy was observed in 79 (40%), 58 (46%), and 41 (55%) patients in the lean, overweight, and obese group (p value = .069). Hematologic toxicity was observed in 31 (16%), 10 (8%), and 4 (5%) in the respective BMI categories, with a significant difference (p value = .025). In 39 cases, the patient experienced neutropenia as hematologic toxicity. Other hematologic toxicities observed were granulocytopenia, leukopenia, anemia, and thrombocytopenia. Other toxicities leading to treatment modifications were infection, edema, and other diverse adverse events. These other toxicities were observed in 21 (10%), 23 (18%), and 12 (16%) patients in the respective BMI categories (p value = .104), in which no apparent differences in toxicity profile were detected between the BMI categories.

Within each BMI category, there were patients who received a RDI < 50%. In lean, overweight, and obese group, this was the case for 6 (3%), 6 (5%), and 7 (9%) patients. In the lean group, this was due to neutropenia (n = 4), peripheral neuropathy (n = 1), and granulocytopenia (n = 1). In the overweight group, this was due to peripheral neuropathy (n = 3), an allergic reaction (n = 1), bad tolerance to the chemotherapy (n = 1), and overall worsening of the health condition (n = 1). In the obesity group, this was due to peripheral neuropathy (n = 5), hepatic impairment in combination with other adverse events (n = 1), and the patient’s own decision to discontinue treatment (n = 1).

### Pathological response

Of the study population, 252 (63%) patients received paclitaxel as neoadjuvant chemotherapy; 128 (51%) lean patients, 78 (31%) overweight patients, and 46 (18%) obese patients. Patient characteristics for this subpopulation are shown in Table 3. Median RDI received were 94%, 90%, and 88% for the lean, overweight, and obese group. Pathological complete response was observed in 22 (17%), 11 (14%), and 6 (13%) patients in the respective BMI categories (p value = .799).

**Table 3:** Characteristics of patients divided by BMI category who received neoadjuvant chemotherapy

	Lean n = 128 (%)	Overweight n = 78 (%)	Obese n = 46 (%)	p value
Age, years				
Mean (SD)	48.1 (11.6)	54.0 (10.7)	53.6 (10.6)	< .001
Tumor size, cm				
< 2	44 (34)	19 (24)	16 (35)	.271
≥ 2	84 (66)	59 (76)	30 (65)	
No. of positive nodes				
0	64 (50)	38 (49)	17 (37)	.496
1–3	56 (44)	36 (46)	24 (52)	
≥ 4	8 (6)	4 (5)	5 (11)	
ER status				
Negative	7 (5)	6 (8)	3 (7)	.786
Positive	121 (95)	72 (92)	43 (94)	
PgR status				
Negative	39 (30)	32 (41)	11 (24)	.125
Positive	89 (70)	46 (59)	35 (76)	
HER2 status				
Negative	101 (79)	65 (83)	40 (87)	.453
Positive	27 (21)	13 (17)	6 (13)	
Histologic grade				
1	8 (6)	3 (4)	2 (4)	.843
2	74 (58)	44 (56)	24 (52)	
3	42 (33)	29 (37)	20 (44)	
Unknown	4 (3)	2 (3)	–	
Ki-67				
< 15%	46 (36)	27 (35)	10 (22)	.361
≥ 15%	76 (59)	49 (63)	33 (72)	
Unknown	6 (5)	2 (2)	3 (6)	
Smoking status				
Never	71 (56)	50 (64)	27 (59)	.261
Ever	57 (45)	28 (36)	18 (39)	
Unknown	–	–	1 (2)	
Menopausal status				
Premenopausal	81 (63)	34 (44)	26 (57)	.022
Postmenopausal	47 (37)	44 (56)	20 (44)	
Pathological response*				
Complete	22 (17)	11 (14)	6 (13)	.799
Near complete	31 (24)	13 (17)	13 (28)	
Major	46 (36)	26 (33)	17 (37)	
Minor	26 (20)	24 (31)	10 (22)	
Unknown	3 (2)	4 (5)	–	

*Complete response, no residual tumor cells; near complete response, < 10% residual tumor cells; major response, 10–50% residual tumor cells; and minor response, > 50% residual tumor cells

## Discussion

This study investigated whether dose capping still occurred in clinical practice and what the influence of BMI was on the tolerability and effectiveness of full-weight-based paclitaxel chemotherapy in early breast cancer patients. None of the patients had their chemotherapy doses capped due to excess weight. There were only small differences observed in the administered RDI of paclitaxel chemotherapy, which were not of clinical significance, in contrast to the results of the study of Desmedt et al. for docetaxel chemotherapy [14]. In a recent study, no significant difference in pharmacokinetic parameters for paclitaxel was observed between lean, overweight, and obese patients, supporting the results in our study [16]. In contrast, the volume of distribution and half-life of docetaxel were significantly increased in obese patients [17].

Although no significant differences were observed in administered RDIs between the different BMI categories, there were differences observed in the type of toxicity leading to chemotherapy treatment modifications. Various studies have already shown that a higher BMI, higher fat mass, or lower skeletal muscle mass are associated with an increased risk and severity of toxicity [18–20]. In line with these results, peripheral neuropathy leading to treatment modifications was observed more frequent in obese patients, although not statistically significant. From other studies, it appeared that the risk of paclitaxel-induced peripheral neuropathy is higher in older patients with estrogen decline [21, 22]. As the patients in the obese group in our study were relatively older and more often postmenopausal, these observations may be related to one another.

The risk of hematologic toxicities decreased significantly with an increasing BMI in our study. Studies investigating toxicity of chemotherapy in breast cancer patients show different results as some found no difference, a lower risk, or even a higher risk of hematologic toxicity in obese patients compared to lean patients [5, 23–25]. It has to be noted that chemotherapy regimens in these studies differed, which may explain the differences observed in these studies. A potential explanation for the decreased risk of hematologic toxicity in obese patients is the presence of chronic inflammation leading to increased neutrophil counts [26–28].

An exploratory analysis of the NeoALTTO trial reported lower pCR rates in overweight and obese patients with hormone-positive and Her2+ breast cancer, compared to underweight and lean patients, whereas this trend was not observed in patients with hormone-negative and Her2+ breast cancer [29]. Wang et al. performed a meta-analysis investigating the impact of BMI on pCR after neoadjuvant chemotherapy in early breast cancer patients. They found that overweight and obese breast cancer patients had lower pCR rates compared to underweight or lean patients. However, in some of the included studies dose capping was performed, whereas for seven of the thirteen included studies, no information on chemotherapy dosing was provided [30]. One of the included studies in this meta-analysis showed that a lower RDI of taxanes resulted in a lower pCR rate [31]. In another study performed by Farr et al., obese women receiving full-weight based chemotherapy even had increased pCR rates and a more favorable progression-free survival compared to lean and overweight patients [32]. In our study, no significant differences were observed in pCR between the BMI categories, which is potentially due to the fact that there were no significant differences in administered RDI between BMI categories.

The main limitation of this study is its retrospective approach, making us dependent on the information that is captured in the electronic medical records. For example, the grading of the toxicity leading to chemotherapy dose reductions, dose delays, or cancellations was often absent. However, the specific adverse events leading to chemotherapy dose modifications was well documented. Another limitation is the relatively low number of obese patients in our study, which is in particular the case in the analysis of pathological response. In addition, BMI may not be the most optimal measure to determine toxicity and survival, as it does not distinguish between muscle and fat mass [18, 19, 33]. Therefore, further research on the influence of obesity on chemotherapy treatment, in which fat mass is determined by CT scans, would be of interest.

In conclusion, BMI did not significantly influence the tolerability and effectiveness of full-weight-based paclitaxel chemotherapy. Therefore, the results of this study align with current guideline recommendations of using full-weight-based paclitaxel chemotherapy in obese patients.

## Data Availability

The dataset generated during the current study are available from the corresponding author on reasonable request.

## References

[1] World Health Organization. Obesity and overweight 2020. https://www.who.int/news-room/fact-sheets/detail/obesity-and-overweight. Accessed 1 Apr 2021

[2] MacMahon S, Baigent C, Duffy S, Rodgers A, Tominaga S, Chambless L (2009). Body-mass index and cause-specific mortality in 900 000 adults: collaborative analyses of 57 prospective studies. Lancet.

[3] Smit C, De Hoogd S, Brüggemann RJM, Knibbe CAJ (2018). Obesity and drug pharmacology: a review of the influence of obesity on pharmacokinetic and pharmacodynamic parameters. Expert Opin Drug Metab Toxicol.

[4] Griggs JJ, Sorbero MES, Lyman GH (2005). Undertreatment of obese women receiving breast cancer chemotherapy. Arch Intern Med.

[5] Lote H, Sharp A, Redana S, Papadimitraki E, Capelan M, Ring A (2016). Febrile neutropenia rates according to body mass index and dose capping in women receiving chemotherapy for early breast cancer. Clin Oncol.

[6] Schwartz J, Toste B, Dizon DS (2009). Chemotherapy toxicity in gynecologic cancer patients with a body surface area (BSA) > 2 m2. Gynecol Oncol.

[7] Barrett SV, Paul J, Hay A, Vasey PA, Kaye SB, Glasspool RM (2008). Does body mass index affect progression-free or overall survival in patients with ovarian cancer? Results from SCOTROC I trial. Ann Oncol Off J Eur Soc Med Oncol.

[8] Madarnas Y, Sawka CA, Franssen E, Bjarnason GA (2001). Are medical oncologists biased in their treatment of the large woman with breast cancer?. Breast Cancer Res Treat.

[9] Wright JD, Tian C, Mutch DG, Herzog TJ, Nagao S, Fujiwara K (2008). Carboplatin dosing in obese women with ovarian cancer: a Gynecologic Oncology Group study. Gynecol Oncol.

[10] Abdah-Bortnyak R, Tsalic M, Haim N (2003). Actual body weight for determining doses of chemotherapy in obese cancer patients: evaluation of treatment tolerability. Med Oncol.

[11] Colleoni M, Li S, Gelber RD, Price KN, Coates AS, Castiglione-Gertsch M (2005). Relation between chemotherapy dose, oestrogen receptor expression, and body-mass index. Lancet.

[12] Griggs JJ, Mangu PB, Temin S, Lyman GH (2012). Appropriate chemotherapy dosing for obese adult patients with cancer: American Society of Clinical Oncology Clinical Practice guideline. J Oncol Pract.

[13] Griggs JJ, Bohlke K, Balaban EP, Dignam JJ, Hall ET, Harvey RD (2021). Appropriate systemic therapy dosing for obese adult patients with cancer: ASCO Guideline update. J Clin Oncol.

[14] Desmedt C, Fornili M, Clatot F, Demicheli R, de Bortoli D, Di Leo A (2020). Differential benefit of adjuvant docetaxel-based chemotherapy in patients with early breast cancer according to baseline body mass index. J Clin Oncol.

[15] DuBois D, DuBois E (1916). A formula to estimate the appropriate surface area if height and weight be known. Arch Intern Med.

[16] Gota V, Nookala M, Bonda A, Karanam A, Shriyan B, Kembhavi Y (2021). Effect of body mass index on pharmacokinetics of paclitaxel in patients with early breast cancer. Cancer Med.

[17] Sparreboom A, Wolff AC, Mathijssen RHJ, Chatelut E, Rowinsky EK, Verweij J (2007). Evaluation of alternate size descriptors for dose calculation of anticancer drugs in the obese. J Clin Oncol.

[18] van den Berg M, Kok D, Posthuma L, Kamps L, Kelfkens C, Buist N (2019). Body composition is associated with risk of toxicity-induced modifications of treatment in women with stage I-IIIB breast cancer receiving chemotherapy. Breast Cancer Res Treat.

[19] Shachar S, Deal A, Weinberg M, Williams G, Nyrop K, Popuri K (2017). Body composition as a predictor of toxicity in patients receiving anthracycline and taxane-based chemotherapy for early-stage breast cancer. Clin Cancer Res.

[20] Timmins HC, Li T, Goldstein D, Trinh T, Mizrahi D, Harrison M (2021). The impact of obesity on neuropathy outcomes for paclitaxel- and oxaliplatin-treated cancer survivors. J Cancer Surviv.

[21] Ghoreishi Z, Keshavarz S, Asghari Jafarabadi M, Fathifar Z, Goodman K, Esfahani A (2018). Risk factors for paclitaxel-induced peripheral neuropathy in patients with breast cancer. BMC Cancer.

[22] Miyamoto T, Hiramoto S, Kanto A, Tsubota M, Fujitani M, Fukuyama H (2021). Estrogen decline is a risk factor for paclitaxel-induced peripheral neuropathy: clinical evidence supported by a preclinical study. J Pharmacol Sci.

[23] Carroll J, Protani M, Nguyen L, Cheng M, Fay M, Saleem M (2014). Toxicity and tolerability of adjuvant breast cancer chemotherapy in obese women. Med Oncol.

[24] Morrison V, McCall L, Muss H, Jatoi A, Cohen H, Cirrincione C (2018). The impact of actual body weight-based chemotherapy dosing and body size on adverse events and outcome in older patients with breast cancer: results from Cancer and Leukemia Group B (CALGB) trial 49907 (Alliance A151436). J Geriatr Oncol.

[25] Furlanetto J, Eiermann W, Marmé F, Reimer T, Reinisch M, Schmatloch S (2016). Higher rate of severe toxicities in obese patients receiving dose-dense (dd) chemotherapy according to unadjusted body surface area: results of the prospectively randomized GAIN study. Ann Oncol Off J Eur Soc Med Oncol.

[26] Furuncuoǧlu Y, Tulgar S, Dogan AN, Cakar S, Tulgar YK, Cakiroglu B (2016). How obesity affects the neutrophil/lymphocyte and platelet/lymphocyte ratio, systemic immune-inflammatory index and platelet indices: a retrospective study. Eur Rev Med Pharmacol Sci.

[27] Kolb R, Sutterwala FS, Zhang W (2016). Obesity and cancer: inflammation bridges the two. Curr Opin Pharmacol.

[28] Orlandini LF, Pimentel FF, de Andrade JM, Dos Reis FJC, de Mattos-Arruda L, Tiezzi DG (2021). Obesity and high neutrophil-to-lymphocyte ratio are prognostic factors in non-metastatic breast cancer patients. Braz J Med Biol Res.

[29] Di Cosimo S, Porcu L, Agbor-tarh D, Cinieri S, Franzoi MA, De Santis MC (2020). Effect of body mass index on response to neo-adjuvant therapy in HER2-positive breast cancer: an exploratory analysis of the NeoALTTO trial. Breast Cancer Res.

[30] Wang H, Zhang S, Yee D, Basu S, Beckwith H, Potter D (2021). Impact of body mass index on pathological complete response following neoadjuvant chemotherapy in operable breast cancer: a meta-analysis. Breast Cancer.

[31] Fontanella C, Lederer B, Gade S, Vanoppen M, Blohmer JU, Costa SD (2015). Impact of body mass index on neoadjuvant treatment outcome: a pooled analysis of eight prospective neoadjuvant breast cancer trials. Breast Cancer Res Treat.

[32] Farr A, Stolz M, Baumann L, Bago-Horvath Z, Oppolzer E, Pfeiler G (2017). The effect of obesity on pathological complete response and survival in breast cancer patients receiving uncapped doses of neoadjuvant anthracycline-taxane-based chemotherapy. Breast.

[33] Cespedes Feliciano E, Chen W, Lee V, Albers K, Prado C, Alexeeff S (2020). Body composition, adherence to anthracycline and taxane-based chemotherapy, and survival after nonmetastatic breast cancer. JAMA Oncol.

